# Association between Systemic Factors and Vitreous Fluid Cytokines in Proliferative Diabetic Retinopathy

**DOI:** 10.3390/jcm12062354

**Published:** 2023-03-17

**Authors:** Tomohito Sato, Rina Okazawa, Koichi Nagura, Hideaki Someya, Yoshiaki Nishio, Toshio Enoki, Masataka Ito, Masaru Takeuchi

**Affiliations:** 1 Department of Ophthalmology, National Defense Medical College, 3-2 Namiki, Tokorozawa 359-8513, Japan; 2 Enoki Eye Clinic, Sayama 350-1316, Japan; 3 Department of Developmental Anatomy and Regenerative Biology, National Defense Medical College, Tokorozawa 359-8513, Japan

**Keywords:** blood pressure, body mass index, influence, interleukin-6, monocyte chemoattractant protein-1, proliferative diabetic retinopathy, vascular endothelial growth factor, vitreous fluid cytokine

## Abstract

Proliferative diabetic retinopathy (PDR) is a vision-threatening complication of diabetes mellitus (DM). Systemic and intraocular factors are intricately related to PDR, and vitreous fluid (VF) cytokines are representative intraocular biomarkers. However, the associations between systemic factors and VF cytokines and their influence on PDR pathology are unclear. This study aimed to examine the correlation between systemic factors and VF cytokines and analyze their contributions to the pathology of PDR using multivariate analyses. We conducted a retrospective observational study on 26 PDR eyes of 25 patients with type 2 DM, and 30 eyes of 30 patients with idiopathic macular hole or epiretinal membrane as controls. Fifteen systemic and laboratory tests including blood pressure (BP) and body mass index (BMI), and 27 cytokines in VF were analyzed. BP and BMI correlated positively with VF levels of IL-6 and IP-10 in PDR patients, while no significant correlation was found between systemic factors and VF cytokines in controls. MCP-1 and VEGF-A in VF separately clustered with different systemic factors in controls, but these cytokines lost the property similarity with systemic factors and acquired property similarity with each other in PDR. Systemic factors contributed to only 10.4%, whereas VF cytokines contributed to 42.3% out of 52.7% variance of the whole PDR dataset. Our results suggest that intraocular factors play a major role in the pathology of PDR, whereas systemic factors may have limited effects, and that BP and BMI control in PDR could be useful interventions to improve intraocular immune condition.

## 1. Introduction

Diabetes mellitus (DM) is a metabolic disease characterized by absolute or relative insulin deficiency [1]. Diabetic retinopathy (DR) is a major complication of DM, and is potentially vision-threatening in DM patients aged 20 to 75 years [2]. DR is characterized by retinal microvascular damage leading to vascular leakage and ischemia-induced retinal neovascularization [3,4]. Numerous etiologic factors potentially involved in the onset and progression of DR have been investigated, including hypertension, obesity, blood glucose level, glycated hemoglobin (Hb)A1c, hyperlipidemia, dietary style, exercise, and smoking [5,6]. Proliferative DR (PDR) is an advanced stage of DR, in which vitreous hemorrhage (VH) and tractional retinal detachment (TRD) occur due to proliferative membrane traction [7,8].

Cytokines are small and nonstructural proteins secreted by a variety of immune and non-immune cells, playing a key role in immune responses in various cells [9,10]. Intraocular fluids comprising aqueous humor (AH) and vitreous fluid (VF) are unique specimens used for the direct analysis of intraocular immune conditions in various chorioretinal diseases such as age-related macular degeneration (AMD) [11,12], uveitis [13,14] and PDR [15,16,17]. In PDR eyes, VF levels of inflammatory cytokines including interleukin (IL)-6, interferon gamma-induced protein 10 (IP-10), monocyte chemotactic protein 1 (MCP-1) and vascular endothelial growth factor (VEGF)-A are elevated [15,16,17].

DR develops through complex interactions among various systemic and intraocular factors [5,18]. Previous studies on DR pathogenesis mainly concentrated on ocular morphological changes observed on color fundus photographs (CFP) and optical coherence tomography (OCT) images. The purposes of this study were to examine the correlations between systemic factors and VF cytokines and to analyze their contributions to the pathology of PDR.

## 2. Materials and Methods

### 2.1. Subjects

This retrospective observational study was performed on 26 PDR eyes of 25 patients with type 2 DM who underwent pars plana vitrectomy (PPV) for VH and/or TRD in National Defense Medical College between 1 January 2014 and 31 August 2021. PDR was diagnosed according to the international clinical disease severity classification of DR [8]. The control group was composed of 14 idiopathic macular hole (MH) eyes of 14 patients, and 16 idiopathic epiretinal membrane (ERM) eyes of 16 patients. The inclusion criteria were [17,19]: (1) no current or past history of intraocular inflammatory diseases including retinal artery occlusion, retinal vein occlusion, AMD, ocular tumor, uveitis, endophthalmitis, and dialysis therapy for renal failure; (2) no history of previous pars plana vitrectomy (PPV), ocular trauma, and prior intravitreal therapies including steroid and anti-VEGF agents such as bevacizumab; and (3) no history of cataract surgery performed within 6 months before the date of enrollment. In one patient with bilateral PDR, both eyes were analyzed separately. When PPV was performed on both eyes in the control group, the VF specimens collected from the first operation were used. The disposition of PDR patients is summarized in Figure S1.

In the PDR group, panretinal photocoagulation was not performed within 7 days before PPV (per inclusion criterion), but was performed more than 7 days before PPV in 16 eyes (61.5%). Twenty-three eyes (88.5%) had VH-obscuring fundus findings. DME and TRD developed in 21 eyes (80.8%) and 16 eyes (61.5%), respectively. DME was defined as retinal edema in the central 3 mm circle on the Early Treatment Diabetic Retinopathy Study (ETDRS) grid [20] in the macula, measured by spectral-domain OCT (SD-OCT; Cirrus HD-OCT, Carl Zeiss Meditec, Dublin, CA, USA). DME and TRD were confirmed by CFP, fluorescein fundus angiography (FAG) and SD-OCT before PPV. When dense VH impeded SD-OCT examination before PPV, the examination was conducted during PPV using an intraoperative OCT system (EnFocus, Leica Microsystems/Bioptigen, Morrisville, NC). In all cases, TRD did not occur within the central 6 mm circle on the ETDRS grid in the macula.

In the present study, we performed a priori power calculation using previous clinical data [17]. We calculated effect sizes (Hedges’ g) for VF IL-6 concentration, which is a representative cytokine with a significant difference between PDR and non-DR control (PDR patients; *n* = 27, IL-6 = 658.5 ± 402.1 pg/mL, ERM patients as control; *n* = 27, IL-6 = 45.7 ± 54.9) [17]. The effect size for VF IL-6 was 2.14. To demonstrate significant differences in VF IL-6 concentration with a statistical power of 0.80 [21], the sample sizes of our study should be at least 4.3 for two-tailed tests. On the other hand, the number of samples used in multivariate analyses should be one or more than that of the explanatory variables [22]. From our preliminary study, 23 variables comprising 11 systemic factors and 12 VF cytokines were expected to be used as explanatory variables in multivariate analyses. Therefore, we attempted to include 25 or more cases each for the PDR and control groups, allowing margin of error.

### 2.2. Diagnostics

PDR, MH and ERM were diagnosed based on a full ophthalmological examination including best-corrected visual acuity (BCVA) test using a decimal chart, intraocular pressure measurement, slit-lamp biomicroscopy, dilated fundus examination, and SD-OCT. Furthermore, CFP and FAG were performed in PDR patients. BCVA was converted to a logarithm of the minimum angle of resolution units (logMAR VA) for statistical analysis. Counting fingers, hand motion, light perception and no light perception were converted to 1.85, 2.30, 2.80 and 2.90 logMAR, respectively [23,24]. Central retinal thickness (CRT) was defined as the mean retinal thicknesses in the central 1 mm circle on the ETDRS grid in the macula [11,12] measured by SD-OCT.

Three retinal specialists (members of Japanese Retina and Vitreous Society) confirmed the diagnoses and reviewed the clinical findings. In case of a discrepancy among the three assessors, the decision was adjudicated by majority rule.

### 2.3. Systemic Factors

Health condition factors of age, body mass index (BMI), systolic blood pressure (SBP), diastolic blood pressure (DBP), pulse pressure difference between SBP and DBP (PPD) and heart rate (HR) were collected at admission for PPV. Systemic condition factors comprising the international normalized ratio of prothrombin time (PT-INR), activated partial thromboplastin time (APTT), random blood glucose (RBG), glycated hemoglobin (Hb) A1c, blood urea nitrogen (BUN), creatinine, estimated glomerular filtration rate (eGFR), C-reactive protein (CRP), urinary glucose and urinary protein were measured at the time of decision to perform PPV. Estimated GFR was calculated using the following formula [25]: 194 × serum creatinine^−1.094^ × age^−0.287^. CRP values lower than 0.3 mg/dL were treated as 0 in statistical analysis. Notation of ± in urine test was assigned a value of 0.5.

### 2.4. Vitreous Fluid Sample Collection and Cytokine Assay

Approximately 0.5 mL of undiluted VF was collected using a 25G vitreous cutter inserted into the mid-vitreous cavity at the beginning of PPV before active infusion [17,19]. Uncentrifuged VF samples were transferred into sterile tubes and stored at −80 °C until processing. No complication associated with VF sampling was observed. Before analysis, undiluted VF samples were centrifuged at 10,000× *g* for 10 min, and 50 μL of the supernatant was used in cytokine assay [17,19]. All standards and samples were assayed in duplicate.

A multiplex bead analysis platform (Bio-Plex Suspension Array System; Bio-Rad) and a multiplex cytokine panel (Bio-Plex Human Cytokine 27-plex panel; Bio-Rad, Hercules, CA, USA) that provides comprehensive coverage of inflammatory mediators were used to detect 27 VF cytokines comprising platelet-derived growth factor-BB, interleukin (IL)-1β, IL-1 receptor antagonist (ra), IL-2, IL-4, IL-5, IL-6, IL-7, IL-8, IL-9, IL-10, IL-12, IL-13, IL-15, IL-17A, eotaxin, basic fibroblast growth factor, granulocyte colony-stimulating factor (G-CSF), granulocyte macrophage colony-stimulating factor, interferon-gamma (IFN-γ), IP-10, MCP-1, MIP (macrophage inflammatory protein)-1α, MIP-1β, regulated on activation, normal T-cell expressed and secreted (RANTES), tumor necrosis factor alpha (TNFα) and VEGF-A. The levels of VF cytokines below detectable levels were treated as 0 in statistical analysis [11,12,17,19].

### 2.5. Statistical Analysis

Statistical analyses were performed using the statistic add-in software for Excel (BellCurve for Excel^®^, SSRI Co., Ltd., Tokyo, Japan, and XLSTAT^®^, Addinsoft company, Paris, France). Data are expressed as mean ± standard deviation [12,17,19]. Mann–Whitney U test and Spearman’s rank correlation test were used for nonparametric comparison and correlation, respectively, between two unpaired groups. Pearson’s chi-squared test (for *n* ≥ 4) and Fisher’s exact test (for *n* < 4) were used to compare categorical variables. Cytokines with detection rates over 60% and high concentrations of 10 pg/mL or above in PDR group were selected as explanatory variables in hierarchical cluster analysis, principal component analysis (PCA), and Spearman’s rank correlation test [11,12]. Hierarchical cluster analysis was performed using Ward’s method with Euclidean distance as the distance metric [11,12]. A two-tailed *p* value less than 0.05 was considered to be statistically significant.

In subsequent multivariate analyses, the number of samples must be one or more than the number of explanatory variables [22]. Therefore, the systemic factors used in the analyses were carefully selected by the following criteria: (1) the systemic factor is recognized as a DR-related factor [6,26,27]; (2) when multiple systemic factors shared the same purpose of evaluation, the factor that provided the most appropriate evaluation was selected; and (3) the systemic test value should be quantitative on a proportional scale. Based on these criteria, age, BMI, SBP, DBP, PPD, HR, PT-INR, APTT, RBG level, HbA1c% and eGFR were chosen as appropriate explanatory variables in subsequent analyses.

## 3. Results

### 3.1. Subjects

The demographic and clinical data of the PDR and control groups are shown in Table 1. The age was 58.6 ± 13.5 years (range 32–82 years) in the PDR group and 70.7 ± 7.94 years (range 48–82) in the control group. The PDR group was significantly younger than the control group, similar to previous studies [17,28]. The Male/female ratio was 18/7 in the PDR group and 13/17 in the control group. LogMAR VA was higher and CRT was lower in the PDR group than in the control group. RBG, HbA1c, BUN, serum creatinine, eGFR, urine glucose and urine protein were higher in the PDR group than in the control group. There were no significant differences in BMI, SBP, DBP, PPD, HR, PT-INR, APTT and CRP between the two groups.

### 3.2. Vitreous Fluid Cytokine Levels

The profiles of VF cytokine levels in the PDR and control groups are summarized in Table 2. The VF levels of IL-1ra, IL-6, IL-7, IL-8, IL-13, IL-15, eotaxin, G-CSF, IFN-γ, IP-10, MCP-1, MIP-1α, MIP-1β, RANTES, TNFα and VEGF-A were higher in PDR group than in control group. Furthermore, IL-1ra, IL-6, IL-7, IL-8, IL-13, eotaxin, IFN-γ, IP-10, MCP-1, MIP-1β, TNFα and VEGF-A had detection rates with higher than 60% and high concentrations of 10 pg/mL or above in the PDR group, and were also detectable in the control group. These 12 VF cytokines that were reliably detectable and had reasonably high concentrations were considered appropriate as explanatory variables in subsequent multivariate analyses [11].

In the subgroup analysis of VF cytokines between TRD and VH eyes in PDR group, there was no significant difference with the levels of all VF cytokines measured (Tables S1 and S2).

### 3.3. Correlation between Systemic Factors and Vitreous Fluid Cytokines

Matrices of *p* values obtained from Spearman’s rank correlation test between systemic factors and VF cytokines are shown in Table 3. In control group (Table 3), positive correlations were found between DBP and HR, and between PT-INR and APTT. SBP correlated positively with DBP, PPD and HR. BMI had negative correlation with APTT, and DBP correlated negatively with RBG and HbA1c. Regarding correlations between VF cytokines, significant correlations were observed among IL-1ra, IL-6, IL-7, IL-8, eotaxin, IFN-γ, IP-10, MCP-1 and MIP-1β. No significant correlation was found between systemic factors and VF cytokines.

In the PDR group (Table 3), positive correlations were observed between age and PPD, and between PT-INR and APTT. SBP correlated positively with BMI, DBP, PPD and RBG. Age correlated negatively with DBP, and PT-INR correlated negatively with RBG. Concerning correlations between systemic factors and VF cytokines, BMI correlated positively with IL-6 and IP-10. Positive correlations were found between SBP and IL-6, IL-7, IL-13, eotaxin or IP-10, and between DBP and IL-6, eotaxin or IP-10. IL-1ra correlated negatively with BMI. Regarding correlations between VF cytokines, all cytokines correlated with one another. In particular, VEGF-A correlated positively with IL-7, IL-13, eotaxin and TNFα. These results suggest that the correlation between systemic factors and VF cytokines could be a pathological feature of PDR. Spearman’s rank correlation coefficients between systemic factors and VF cytokines are presented in Table S3.

### 3.4. Expression Patterns of Systemic Factors and Vitreous Fluid Cytokines in Hierarchical Cluster Analysis

**Figure 1:**
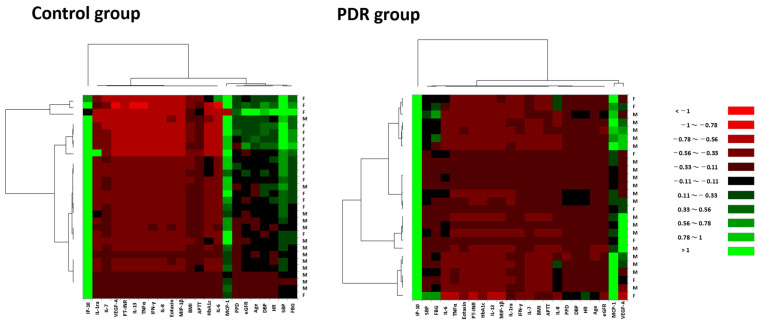
Hierarchical cluster analysis showing expression patterns of systemic factors and vitreous fluid cytokines in PDR patients and controls. Heat maps are composed of age, BMI, SBP, DBP, PPD, HR, PT-INR, APTT, RBG, HbA1c and eGFR as systemic factors; and vitreous fluid cytokines comprising IL-1ra, IL-6, IL-7, IL-8, IL-13, eotaxin, IFN-γ, IP-10, MCP-1, MIP-1β, TNFα and VEGF-A. Color scale: low values, red; middle to high values, black to green. Vertical axis indicates gender of subjects, and horizontal axis shows explanatory variables.

Cluster analysis was performed to classify systemic factors and VF cytokines into groups with relatively similar properties called clusters [29] (pp. 603–16). In the control group (Figure 1), hierarchical cluster analysis classified the explanatory variables broadly into single cytokine and two principal clusters as follows: (1) the single cytokine was IP-10; (2) one cluster consisted of one cytokine MCP-1 and a subcluster formed by age, SBP, DBP, PPD, HR, RBG and eGFR; and (3) another cluster was composed of BMI, PT-INR, APTT, HbA1c and the remaining cytokines including VEGF-A.

In the PDR group (Figure 1), the explanatory variables were roughly classified into single cytokine and two principal clusters as follows: (1) the single cytokine was IP-10; (2) one cluster consisted of two cytokines, MCP-1 and VEGF-A; and (3) another cluster was composed of all the systemic factors and the remaining cytokines. In summary, although MCP-1 and VEGF-A clustered independently with different systemic factors in control group, the two cytokines were altered in the PDR group, losing property similarity with the systemic factors and acquiring property similarity with each other, independent of the influences of systemic factors.

### 3.5. Principal Component Analysis for Expression Patterns of Systemic Factors and Vitreous Fluid Cytokines in Controls

**Figure 2:**
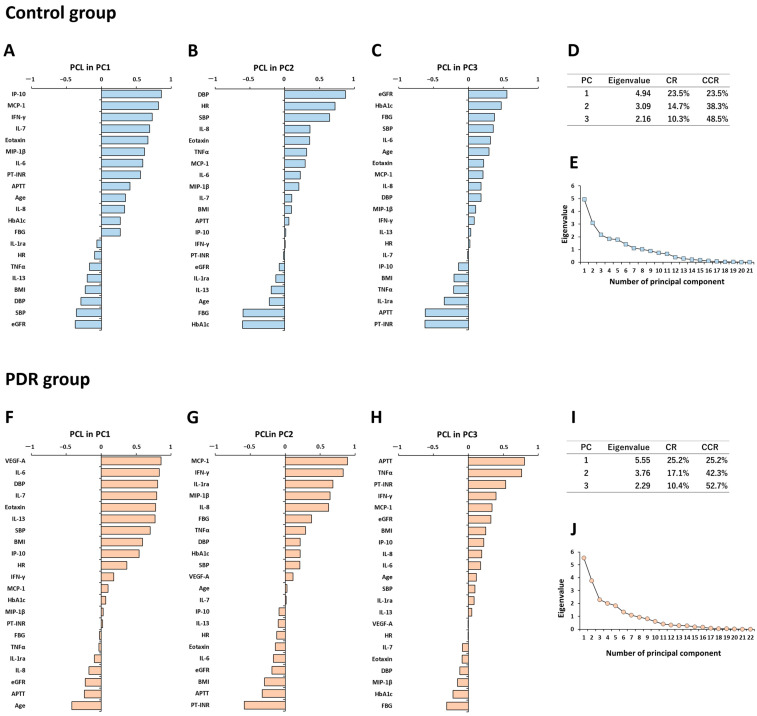
Principal component analysis showing expression patterns of systemic factors and vitreous fluid cytokines in PDR patients and controls. Expression patterns of systemic factors and vitreous fluid cytokines in PCA are shown in (**A**–**E**) controls and (**F**–**J**) PDR patients. In PCA of control group, PCLs of (**A**) PC1, (**B**) PC2 and (**C**) PC3 are shown. (**D**) eigenvalue, CR and CCR, and (**E**) plots of eigenvalue of all PCs are presented. In PDR group, PCLs of (**F**) PC1, (**G**) PC2 and (**H**) PC3 are shown. (**I**) eigenvalue, CR and CCR, and (**J**) plots of eigenvalue of all PCs are presented. CCR; cumulative contribution ratio, CR; contribution ratio, PC; principal component, PCA; principal component analysis, PCL; principal component loading, PC1; first principal component, PC2; second principal component, PC3; third principal component.

In the PCA of the control group, the variance of VEGF-A was less than 1.0 × 10^−10^, and PPD showed multicollinearity with other explanatory variables. Therefore, VEGF-A and PPD were excluded from PCA. The contribution rates (CRs) of the first (PC1), second (PC2) and third principal component (PC3) were, respectively, 23.5%, 14.7% and 10.3% (cumulative 48.5%) of the total variance of the entire dataset (Figure 2D).

In the principal component loading (PCL) analysis of PC1 (Figure 2A), IP-10, MCP-1, IFN-γ, IL-7, eotaxin and MIP-1β had high loadings of over 0.6, and the loading of age was approximately 0.35, while eGFR, SBP and DBP had negative loadings. The results suggest that intraocular immune regulation by immunocompetent cells including T cells, macrophages and eosinophils would work in an exquisitely balanced manner, and the cytokines secreted tend to increase with aging, while systemic hemodynamics and renal function generally deteriorate with aging. Therefore, PC1 as a dominant unsupervised summary axis may be interpreted as the primary intraocular immune condition in older healthy subjects.

In the PCL analysis of PC2 (Figure 2B), DBP, HR and SBP had high loadings of over 0.6. HbA1c, RBG and age had negative loadings of −0.61, −0.60 and −0.22, respectively. These results imply that BP and HR decrease, whereas RBG and HbA1c increase with aging. PC2 may be interpreted as the functions of systemic hemodynamics and glucose tolerance.

In the PCL analysis of PC3 (Figure 2C), the loadings of eGFR, HbA1c and RBG were, respectively, 0.55, 0.47 and 0.37, whereas PT-INR and APTT had negative loadings of −0.623 and −0.618. The loading of age was approximately 0.30, suggesting some influence on PC3. PC3 may be interpreted as the relationship among glucose tolerance, renal function and blood coagulability.

Based on the interpretation of each principal component, the cumulative CR of systemic factors dominating PC2 and PC3 was 25.0%, and that of VF cytokines being the major constituents of PC1 was 23.5%, out of 48.5% variance of the whole dataset. The plot of eigenvalues of all principal components are presented in Figure 2E.

### 3.6. Principal Component Analysis for Expression Patterns of Systemic Factors and Vitreous Fluid Cytokines in PDR Patients

In the PCA of the PDR group, PPD was excluded from analysis because of multicollinearity with other explanatory variables. The CRs of PC1, PC2 and PC3 were, respectively, 25.2%, 17.1% and 10.4% (cumulative 52.7%) of the total variance of the dataset (Figure 2I).

In the PCL analysis of PC1 (Figure 2F), VEGF-A and IL-6 had the highest loadings of 0.86 and 0.83, while DBP and SBP also had high loadings (0.81 and 0.70). Age had a negative loading of −0.42. The results suggest that in PDR patients, younger age is associated with higher VF levels of VEGF-A and IL-6 accompanied by BP elevation. PC1 may be interpreted as the primary intraocular immune condition, implying angiogenesis and inflammation in response to retinal ischemia, which is also associated with systemic hemodynamics.

In the PCL analysis of PC2 (Figure 2G), MCP-1, IFN-γ, IL-1ra, MIP-1β and IL-8 had high loadings of over 0.6, whereas PT-INR and APTT had negative loadings (−0.58 and −0.33). The loading of age was only 0.03, indicating no influence on PC2. PC2 may be interpreted as the second major intraocular immune condition, possibly influenced by blood coagulability.

In the PCL analysis of PC3 (Figure 2H), APTT, TNFα and PT-INR had high loadings of 0.80, 0.76 and 0.53, respectively. RBG and HbA1c had negative loadings of −0.31 and −0.22. The loading of age was only 0.12, indicating almost no effect on PC3. The results suggest that the decreases of APTT and PT-INR are accompanied by the elevation of RBG and HbA1c. PC3 may be interpreted as the relationship between glucose tolerance and blood coagulability, similar to PC3 in controls.

Significant variables in PC1 and PC2 mainly consisted of VF cytokines, while those in PC3 were primarily systemic factors. Based on the interpretation of each principal component, the cumulative CR of VF cytokines dominating PC1 and PC2 was 42.3% and that of systemic factors mainly constituting PC3 was 10.4%, out of 52.7% variance of the entire dataset. The eigenvalues of all principal components are plotted in Figure 2J.

## 4. Discussion

In this study, we demonstrated the correlations between systemic factors and VF cytokines as representative intraocular factors in PDR, and comprehensively evaluated the influences of systemic factors and VF cytokines on the pathology of PDR using multivariate analyses. The primary findings of the current study were as follows: (1) BP and BMI correlated positively with VF levels of IL-6 and IP-10 in PDR patients, while there was no significant correlation between systemic factors and VF cytokines in controls; (2) MCP-1 and VEGF-A in the VF independently clustered with different systemic factors in controls, but these cytokines lost the property similarity with systemic factors and acquired property similarity with each other in PDR patients; (3) systemic factors contributed to only 10.4%, whereas VF cytokines contributed to 42.3% out of 52.7% variance of the whole PDR dataset.

A large number of health and systemic conditions including hypertension, obesity, hyperlipidemia, blood glucose, HbA1c as well as dietary style, exercise, and smoking are known to be potentially related to DR [5,6]. In particular, hypertension is consistently associated positively with the development and progression of DR [6,30,31]. For DM, intensive blood glucose control from the early stage was found to suppress the development and progression of DM-related complications over the long-term, a phenomenon termed “legacy effect” [27,32] or “metabolic memory [33,34,35]. Concerning renal function, lower GFR and proteinuria increased the prevalence and progression risk of DR in several clinical studies [36,37,38,39]. A retrospective analysis of non-PDR patients conducted in the United States showed that nephropathy increased the risk of progression to PDR by 29% [40]. Based on the reported DR-related factors, we selected those related to systemic hemodynamics, renal function, glucose tolerance, and anticoagulant activity as systemic factors potentially associated with PDR in this study.

Various intraocular inflammatory and angiogenic factors are involved in the development and progression of DR. The VF levels of IL-6, IL-8, TNFα and VEGF-A were elevated in PDR eyes compared to non-diabetic eyes [15,17,19]. Retinal ischemia increases compensatory angiogenesis, tissue remodeling and inflammation, presumably mediated by elevated expression of IL-6, IL-1𝛽, TNF𝛼 and VEGF [41]. On the other hand, cytokines demonstrate functional multiplicity and diversity by interacting with one another [10,11,42]. Therefore, we conducted a comprehensive analysis of the influences of systemic factors and VF cytokines on the pathology of PDR using PCA, aiming to elucidate the intricately intertwined relationship among those factors.

Correlation existed between systemic factors and VF cytokines in PDR patients, but not in older healthy subjects (Table 3). In addition, BP and BMI were associated with increases or decreases of VF IL-6 and IP-10 levels. IL-6 is involved in low-grade inflammation via immune responses in type 2 DM [43], and directly or indirectly induces numerous angiogenic and inflammatory cytokines including VEGF [44]. IP-10 is a specific chemokine of type 1 helper cells [45], and acts as antiangiogenic and antifibrotic factors [46,47]. We speculate that elevated IP-10 level in the VF may be a homeostatic response for suppressing angiogenesis via low-grade inflammation in PDR. Regarding BP in DR, the UKPDS reported that a 10 mmHg reduction in SBP reduced the risk of developing DR by approximately 10%, and strict BP control decreased DR progression by 35% and vision loss by 47% [27,32]. As for dietary style in DR, several studies suggested that greater adherence to the Mediterranean diet and higher intake of fruit, vegetable and fish may protect against the development of DR [48,49,50]. Therefore, our results support proper management of BP and dietary style as useful systemic interventions to improve intraocular immune conditions in PDR eyes, which is generally consistent with previous studies. On the other hand, VEGF-A in the VF did not correlate with systemic factors or IL-6, implying that the VF level of VEGF-A could not be reduced sufficiently by controlling systemic factors in PDR patients. In real-world practice, intravitreal injection of an anti-VEGF agent induces marked regression of retinal neovascularity secondary to DR, especially in the cases with neovascular glaucoma [51]. Therefore, our results and previous studies suggest that both systemic and topical eye treatments are required for managing PDR.

Cluster analysis is a summary statistical method that classifies explanatory variables into relatively similar groups called clusters [29] (pp. 603–616). Cluster analysis results suggest that the property similarity of systemic factors with VF cytokines was weakened and limited in PDR patients compared to controls. In particular, the property similarities of MCP-1 and VEGF-A in the VF deviated from all systemic factors in PDR, suggesting that these VF cytokines may not be significantly affected by systemic factors. VEGF-A is a crucial mediator of angiogenesis and vascular permeability [52], and recruits macrophages by binding VEGF receptor-1 in the process of inflammatory neovascularization [53]. MCP-1 is a chemokine regulating migration and infiltration of macrophages [54], plays pathogenic roles in DR via low-grade inflammation [55,56], and is also related to vascular permeability [57]. Currently, intravitreal anti-VEGF injection [58], intravitreal triamcinolone acetonide (TA) [59] and sub-Tenon TA [60] are the standard treatments for DME [61]. The present study could provide a rationale that topical ophthalmic treatment is an effective therapy to inhibit the activity of PDR compared to systemic treatment.

PCA is a statistical technique that summarizes quantitative multivariate data by converting many correlated variables into fewer uncorrelated variables called principal components [62] (pp. 1094–1096). In this study, 23 variables comprising 11 systemic factors and 12 VF cytokines were reconstructed into a new unsupervised summary axis according to the order of influence on the pathology of PDR, and the interpretations of the summary axis were based on the magnitude and plus or minus sign of the PCL of explanatory variables constituting that axis. In PDR group, significant variables in PC1 and PC2 were dominantly VF cytokines, with cumulative CR of 42.3%, whereas significant variables in PC3 were primarily systemic factors possibly interpreted as functions of glucose tolerance and blood coagulability, with CR of only 10.4%. It is clinically important to assess whether systemic treatment impacts the pathogenesis of PDR. In the Wisconsin Epidemiologic Study of Diabetic Retinopathy, metabolic controls of HbA1c, BP and total cholesterol, and disease duration accounted for only 11% of the risk of DR in type 1 DM patients, while the remaining 89% was due to other factors [63,64]. DR and DME disease severity scales have been proposed [8], but the influence of systemic factors on the pathology of DR in each stage with or without DME has not been examined in detail. Further research is needed to elucidate the impact of the complex relationship between systemic and intraocular factors on the pathology of DR. Aqueous humor (AH) is an intraocular specimen that can be collected repeatedly and less invasively compared to VF [65], and will be a useful source of biomarkers to explore intraocular immune environment in DR.

In the present study, serum levels of cytokines were not examined. Since the blood-retinal barrier could be disrupted in PDR, serum cytokines may affect the VF cytokine levels. Takeuchi et al. [17] reported a markedly lower serum IL-6 level (2.82 ± 10.2 pg/mL) than VF IL-6 level (658.5 ± 402.1 pg/mL) in PDR patients. In addition, when comparing the levels of VF cytokines between PDR patients with or without VH, there was no significant difference with those of 15 VF cytokines. Nevertheless, the contamination of VF samples by serum cytokines and blood cells is a potential bias, leading to misinterpretation of VF cytokines. A further comprehensive study analyzing complex data composed of AH, VF, blood and systemic factors will help to understand the pathogenesis of PDR.

The present study had several limitations. (1) This study was a retrospective observational study, and the data analyzed were limited to routine examinations and laboratory tests covered by health insurance. Potential DR-related risk factors [5] including disease duration, hyperlipidemia, dietary style, exercise, and smoking were not included due to incomplete medical records. BCVA, CRT and fundus findings were not adopted as explanatory variables in multivariate analyses due to the high prevalence of VH (88.5%) that significantly undermined the reliability of ophthalmic findings. (2) The sample size was small, which limits the generalizability to all types of PDR patients. (3) The PDR group consisted of younger patients compared to the control group, similar to previous studies [17,28]. A relative disease control group was used, not a legitimate control group composed of DM patients with no DR or PDR, because PPV is not performed in such patients and VF specimens are not available. In future, further case–control studies based on a baseline of systemic disease need to be performed. (4) The instability of systemic disease markers as poor controls of systemic treatment could be a potential bias in the interpretation of association between systemic factors and VF cytokines in the pathology of PDR. (5) Hierarchical cluster analysis and PCA are unsupervised analyses, and some degrees of interpretation freedom are allowed. The interpretation criteria are based on the distance (property similarity) and expression intensity (influence) of the sorted explanatory variables in hierarchical cluster analysis, and the order of PC (contribution) and the magnitude of PCL of the PC (property similarity and influence) in PCA. However, the sorting of explanatory variables in those analyses were automatically calculated, and there was no bias in the presented data.

A key strength of our study is that systemic and intraocular PDR-related factors were examined simultaneously, and their complex relations and influences on the pathology of PDR were evaluated comprehensively by multivariate analyses. Furthermore, the data used in this study were obtained from real-world management of PDR, and there was no arbitrary intervention bias in the treatment process.

## 5. Conclusions

The influence of systemic factors on the pathology of PDR may be weakened compared to that of intraocular factors. BP and BMI were important systemic factors that correlate positively with VF levels of inflammatory cytokines. Therefore, in addition to intraocular treatments, BP and BMI managements could be useful interventions to improve intraocular immune conditions. Ophthalmologists and physicians need to be aware of the effectiveness of both systemic and topical eye treatments, and work closely together to prevent the progression of DR, a vision-threatening complication of DM.

## Figures and Tables

**Table 1:** Demographic and clinical data of PDR patients and controls.

Category	PDR		Control		*p* Value	Unit	Reference Range
*n*	26		30				
	Detectable	Mean ± SD	Detectable	Mean ± SD			
	Rate (%)		rate (%)				
**Age**	26 (100)	58.6 ± 13.5	30 (100)	70.7 ± 7.94	**0.001**	year	
Gender (M/F)	18 (69.2)/7		13 (43.3)/17		0.063		
Laterality							
(B/R/L)	1/14/10		0/14/16		0.378		
**LogMAR VA**	26 (100)	1.62 ± 0.72	30 (100)	0.45 ± 0.40	**2.05 × 10^−6^**		
IOP	26 (100)	14.5 ± 3.56	30 (100)	15.0 ± 2.30	0.464	mmHg	10 to 21
**CRT**	5 (19.2)	307.0 ± 67.4	30 (100)	429.2 ± 88.4	**0.015**	μm	
Subgroup							
PRP (+/−)	16 (61.5)/10		−				
Focal PC (+/−)	0 (0)/26		−				
DME (+/−)	21 (80.8)/5		−				
TRD (+/−)	16 (61.5)/10		−				
VH (+/−)	23 (88.5)/3		−				
IVB (+/−)	0 (0)/26		−				
NVG (+/−)	0 (0)/26		−				
MH/ERM	−		14/16				
BMI	26 (100)	24.3 ± 4.76	30 (100)	23.4 ± 4.24	0.455		18.5 to 25.0
SBP	26 (100)	134.5 ± 18.9	30 (100)	139.7 ± 19.7	0.270	mmHg	140 >
DBP	26 (100)	78.6 ± 14.6	30 (100)	79.2 ± 10.2	0.511	mmHg	90 >
PPD	26 (100)	55.8 ± 15.4	30 (100)	60.5 ± 14.3	0.133	mmHg	
HR	26 (100)	78.2 ± 10.5	30 (100)	76.3 ± 14.2	0.376	beats/min	45 to 85
PT-INR	26 (100)	0.97 ± 0.04	30 (100)	1.04 ± 0.30	0.098		0.85 to 1.15
APTT	26 (100)	27.6 ± 2.27	30 (100)	28.7 ± 3.78	0.364	sec	24 to 34
**FBS**	26 (100)	159.4 ± 59.4	30 (100)	102.8 ± 11.6	**8.21 × 10^−6^**	mg/dL	70 to 109
**HbA1c**	26 (100)	7.40 ± 1.47	30 (100)	5.98 ± 0.61	**5.39 × 10^−5^**	%	4.6 to 6.2
**BUN**	26 (100)	22.2 ± 10.6	30 (100)	15.1 ± 3.32	**0.002**	mg/dL	8 to 20
**Cre**	26 (100)	1.28 ± 0.79	30 (100)	0.75 ± 0.21	**0.002**	mg/dL	0.65 to 1.07
**eGFR**	26 (100)	58.4 ± 28.9	30 (100)	69.5 ± 13.1	**0.037**	mL/min/1.73 m^2^	≥60
CRP	26 (100)	0.13 ± 0.31	30 (100)	0.08 ± 0.30	0.450	mg/dL	0.3 >
**U-glucose**	26 (100)	1.38 ± 1.65	30 (100)	0	**0.001**	mg/dL	2 to 20
**U-protein**	26 (100)	1.35 ± 1.16	30 (100)	0.11 ± 0.28	**6.57 × 10^−5^**	mg/dL	0 to 10

APTT; activated partial thromboplastin time, B; bilateral, BMI; body mass index, BUN; blood urea nitrogen, Cre; creatinine, CRP; C-reactive protein, CRT; central retinal thickness, DBP; diastolic blood pressure, DME; diabetic macular edema, eGFR; estimated glomerular filtration rate, ERM; idiopathic epiretinal membrane, F; female, HbA1c; glycated hemoglobin A1c, HR; heart rate, IOP; intraocular pressure, IVB; intravitreal injection of bevacizumab, logMAR; logarithm of the minimum angle of resolution, L; left, M; male, MH; idiopathic macular hole, mmHg; millimeter of mercury, *n*; number, NVG; neovascular glaucoma, PC; photocoagulation, PDR; proliferative diabetic retinopathy, PPD; pulse pressure difference between SBP and DBP, PRP; panretinal photocoagulation, PT-INR; international normalized ratio of prothrombin time, R; right, RBG; random blood glucose, SBP; systolic blood pressure, SD; standard deviation, sec; second, TRD; tractional retinal detachment, U-glucose; urine glucose, U-protein; urine protein, VA; visual acuity, VH; vitreous hemorrhage. Items with significant differences were listed in bold.

**Table 2:** Vitreous fluid levels of cytokines in PDR patients and controls.

Category	PDR		Control		*p* Value	Detection Range		
*n*	26		30					
	Detectable	Mean ± SD	Detectable	Mean ± SD				
	Rate (%)		Rate (%)					
PDGF-BB	8 (30.8)	15.4 ± 42.2	0	0	0.054	1.00	to	52,401
IL-1β	5 (19.2)	0.09 ± 0.21	0	0	0.226	0.06	to	4598
**IL-1ra**	17 (65.4)	23.9 ± 32.8	13 (43.3)	16.3 ± 47.1	**0.034**	2.52	to	247,147
IL-2	1 (3.85)	0.07 ± 0.37	0	0	0.531	0.54	to	26,397
IL-4	9 (34.6)	0.18 ± 0.32	16 (53.3)	0.10 ± 0.12	0.518	0.08	to	4035
IL-5	2 (7.69)	1.24 ± 4.46	0	0	0.513	3.69	to	81,206
**IL-6**	23 (88.5)	133.2 ± 159.1	28 (93.3)	8.53 ± 19.4	**0.001**	0.37	to	21,699
**IL-7**	26 (100)	35.9 ± 30.7	25 (83.3)	17.8 ± 12.2	**0.008**	0.49	to	41,077
**IL-8**	26 (100)	95.7 ± 102.5	22 (73.3)	2.57 ± 3.23	**3.97 × 10^−8^**	0.75	to	27,477
IL-9	6 (23.1)	2.01 ± 4.30	0	0	0.152	0.92	to	45,633
IL-10	6 (23.1)	2.58 ± 6.00	0	0	0.152	0.74	to	23,402
IL-12	7 (26.9)	13.2 ± 27.2	0	0	0.090	1.23	to	21,022
**IL-13**	16 (61.5)	11.1 ± 16.8	3 (10.0)	0.11 ± 0.38	**0.001**	0.32	to	9203
**IL-15**	9 (34.6)	2.77 ± 4.09	0	0	**0.031**	1.62	to	251,038
IL-17A	4 (15.4)	1.05 ± 2.82	0	0	0.330	1.71	to	41,194
**Eotaxin**	23 (88.5)	17.6 ± 13.2	29 (96.7)	5.85 ± 3.34	**0.001**	0.07	to	7488
bFGF	8 (30.8)	9.86 ± 21.4	0	0	0.054	3.02	to	3939
**G-CSF**	12 (46.2)	24.8 ± 59.2	3 (10.0)	1.08 ± 3.32	**0.019**	1.67	to	138,193
GM-CSF	1 (3.85)	0.05 ± 0.26	0	0	0.531	0.38	to	10,559
**IFN-γ**	25 (96.2)	37.1 ± 27.7	27 (90.0)	5.32 ± 4.52	**4.80 × 10^−7^**	0.74	to	20,941
**IP-10**	26 (100)	4568.3 ± 4746.5	30 (100)	979.0 ± 810.6	**8.70 × 10^−6^**	2.75	to	48,834
**MCP-1**	26 (100)	717.4 ± 460.1	30 (100)	209.0 ± 86.1	**7.10 × 10^−7^**	0.44	to	11,213
**MIP-1α**	26 (100)	2.09 ± 1.65	21 (70.0)	0.16 ± 0.13	**7.27 × 10^−8^**	0.05	to	1045
**MIP-1β**	26 (100)	11.5 ± 9.16	27 (90.0)	3.34 ± 2.98	**3.99 × 10^−5^**	0.29	to	6180
**RANTES**	9 (34.6)	8.18 ± 18.0	1 (3.33)	0.09 ± 0.50	**0.042**	1.41	to	7569
**TNFα**	21 (80.8)	15.9 ± 21.3	1 (3.33)	0.15 ± 0.80	**3.83 × 10^−6^**	2.73	to	63,996
**VEGF-A**	22 (84.6)	870.0 ± 1680.8	1 (3.33)	0.16 ± 0.89	**1.55 × 10^−6^**	2.42	to	178,228

bFGF; basic fibroblast growth factor, G-CSF; granulocyte colony-stimulating factor, GM-CSF; granulocyte macrophage colony-stimulating factor, IFN; interferon, IP-10; interferon gamma-induced protein 10, IL; interleukin, MIP; macrophage inflammatory protein, MCP; monocyte chemotactic protein, PDGF; platelet derived growth factor, ra; receptor antagonist, RANTES; regulated on activation, normal T-cell expressed and secreted, TNF; tumor necrosis factor, VEGF; vascular endothelial growth factor. Cytokines with significant differences were listed in bold.

**Table 3:** Correlation matrices with significance levels for systemic factors and vitreous fluid cytokines in PDR patients and controls.

**Control Group**																							
	**Age**	**BMI**	**SBP**	**DBP**	**PPD**	**HR**	**PT-INR**	**APTT**	**RBG**	**HbA1c**	**eGFR**	**IL-1ra**	**IL-6**	**IL-7**	**IL-8**	**IL-13**	**Eotaxin**	**IFN-γ**	**IP-10**	**MCP-1**	**MIP-1β**	**TNFα**	**VEGF-A**
**Age**	−	0.236	0.982	0.591	0.674	0.506	0.114	0.053	0.180	0.462	0.240	0.213	0.546	0.735	0.975	0.094	0.157	0.615	0.060	0.528	0.943	0.093	0.955
**BMI**		−	0.504	0.499	0.513	0.868	0.489	**0.047**	0.988	0.821	0.080	0.989	0.775	0.907	0.755	0.210	0.823	0.618	0.727	0.325	0.756	0.778	0.612
**SBP**			−	**1.14 × 10^−5^**	**4.65 × 10^−9^**	**0.001**	0.557	0.367	0.293	0.129	0.471	0.565	0.323	0.734	0.645	0.980	0.839	0.796	0.422	0.855	0.467	0.866	0.778
**DBP**			**	−	0.085	**3.18 × 10^−4^**	0.894	0.373	**0.008**	**0.022**	0.407	0.393	0.952	0.831	0.317	0.781	0.223	0.874	0.925	0.927	0.933	0.395	0.279
**PPD**			**		−	0.190	0.842	0.737	0.941	0.686	0.281	0.944	0.196	0.774	0.923	0.650	0.722	0.857	0.292	0.703	0.338	0.280	0.735
**HR**			**	**		−	0.278	0.231	0.195	0.111	0.589	0.110	0.824	0.896	0.665	0.229	0.880	0.945	0.993	0.735	0.982	0.462	0.334
**PT-INR**							−	**0.007**	0.379	0.831	0.532	0.170	0.317	0.420	0.097	0.354	0.999	0.806	0.735	0.959	0.626	0.278	0.278
**APTT**		*					**	−	0.800	0.085	0.887	0.240	0.144	0.526	0.862	0.711	0.404	0.342	0.316	0.372	0.509	0.907	0.348
**RBG**				**					−	0.109	0.221	0.763	0.512	0.853	0.708	0.888	0.900	0.127	0.368	0.305	0.918	0.151	0.334
**HbA1c**				*						−	0.902	0.915	0.722	0.501	0.429	0.767	0.678	0.586	0.313	0.462	0.085	0.168	N/A
**eGFR**											−	0.241	0.845	0.952	0.427	0.742	0.597	0.293	0.899	0.265	0.218	0.955	0.280
**IL-1ra**												−	0.342	0.492	0.985	0.994	**0.002**	0.502	**0.017**	0.704	0.504	0.416	0.145
**IL-6**													−	0.174	0.074	0.426	0.158	**0.010**	0.058	**0.002**	**0.030**	0.534	0.094
**IL-7**														−	0.158	0.091	**0.006**	**0.011**	**5.56 × 10^−4^**	**0.049**	0.250	0.611	0.305
**IL-8**															−	0.944	0.202	**0.034**	0.230	0.062	0.616	0.649	0.148
**IL-13**																−	0.803	0.884	0.264	0.202	0.639	0.746	0.746
**Eotaxin**												**		**			−	0.133	**7.60 × 10^−7^**	0.142	**0.035**	0.462	0.121
**IFN-γ**													**	*	*			−	**0.040**	**2.43 × 10^−11^**	**0.017**	0.534	0.611
**IP-10**												*		**			**	*	−	**0.020**	**0.033**	0.612	0.121
**MCP-1**													**	*				**	*	−	**0.004**	0.462	0.189
**MIP-1β**													*				*	*	*	**	−	0.572	0.866
**TNFα**																						−	0.856
**VEGF-A**																							−
**PDR group**																							
	**Age**	**BMI**	**SBP**	**DBP**	**PPD**	**HR**	**PT-INR**	**APTT**	**RBG**	**HbA1c**	**eGFR**	**IL-1ra**	**IL-6**	**IL-7**	**IL-8**	**IL-13**	**Eotaxin**	**IFN-γ**	**IP-10**	**MCP-1**	**MIP-1β**	**TNFα**	**VEGF-A**
**Age**	−	0.060	0.763	**0.002**	**0.029**	0.587	0.534	0.428	0.431	0.192	0.861	0.978	0.261	0.764	0.056	0.462	0.055	0.208	0.295	0.578	0.442	0.231	0.809
**BMI**		−	**0.032**	0.241	0.272	0.564	0.408	0.764	0.775	0.121	0.775	**0.048**	**0.043**	0.069	0.925	0.436	0.428	0.735	**0.025**	0.812	0.714	0.826	0.330
**SBP**		*	−	**0.006**	**0.002**	0.149	0.612	0.404	**0.029**	0.561	0.587	0.959	**0.021**	**0.022**	0.254	**0.031**	**0.039**	0.170	**0.002**	0.187	0.190	0.250	0.213
**DBP**	**		**	−	0.219	0.079	0.293	0.074	0.405	0.454	0.330	0.918	**0.024**	0.056	0.568	0.297	**0.011**	0.129	**0.035**	0.162	0.438	0.173	0.381
**PPD**	*		**		−	0.966	0.890	0.339	0.256	0.428	N/A	0.824	0.589	0.360	0.180	0.162	0.966	0.873	0.204	0.751	0.541	0.907	0.586
**HR**						−	0.740	0.789	0.930	0.160	0.838	0.876	0.071	0.640	0.973	0.822	0.054	0.675	0.060	0.883	0.846	0.188	0.274
**PT-INR**							−	**0.004**	**0.018**	0.469	0.097	0.155	0.572	0.589	0.674	0.140	0.883	0.389	0.694	0.341	0.394	0.396	0.699
**APTT**							**	−	0.232	0.130	0.125	0.717	0.999	0.392	0.739	0.786	0.477	0.651	0.365	0.581	0.507	0.410	0.478
**RBG**			*				*		−	0.138	0.746	0.519	0.849	0.849	0.527	0.475	0.690	0.689	0.660	0.398	0.525	0.932	0.474
**HbA1c**										−	0.532	0.364	0.636	0.697	0.369	0.291	0.614	0.976	0.694	0.904	0.987	0.527	0.158
**eGFR**											−	0.301	0.669	0.648	0.360	0.217	0.619	0.613	0.761	0.966	0.650	0.900	0.635
**IL-1ra**		*										−	0.082	**0.031**	0.136	0.200	0.596	**1.45 × 10^−4^**	0.529	**8.95 × 10^−4^**	0.693	**7.73 × 10^−4^**	0.341
**IL-6**		*	*	*									−	**6.69 × 10^−5^**	0.559	0.075	**0.018**	0.893	**2.95 × 10^−6^**	0.904	0.126	0.673	0.156
**IL-7**			*									*	**	−	0.516	**8.87 × 10^−6^**	0.262	0.485	**0.014**	0.943	**0.046**	0.371	**0.024**
**IL-8**															−	0.108	0.561	**0.022**	0.204	**0.002**	**0.001**	**0.040**	0.313
**IL-13**			*											**		−	0.264	0.921	0.157	0.577	0.080	0.805	**0.002**
**Eotaxin**			*	*									*				−	0.225	**0.006**	0.846	0.946	**0.045**	**0.002**
**IFN-γ**												**			*			−	**0.039**	**5.40 × 10^−9^**	0.124	**2.50 × 10^−7^**	0.268
**IP-10**		*	**	*									**	*			**	*	−	**0.021**	**0.017**	0.159	0.246
**MCP-1**												**			**			**	*	−	**0.003**	**7.70 × 10^−5^**	0.343
**MIP-1β**														*	**				*	**	−	0.702	0.131
**TNFα**												**			*		*	**		**		−	**0.049**
**VEGF-A**														*		**	**					*	−

Numerical data are *p* values computed by Spearman’s rank correlation test. Asterisks denote significant differences. Blue boxes indicate positive correlation, and red boxes denote negative correlation. N/A; not applicable because correlation coefficient is less than 1.0 × 10^−10^, * *p* < 0.05, ** *p* < 0.01.

## Data Availability

The original contributions presented in the study are included in the article/Supplementary Material. Further inquiries can be directed to the corresponding author.

## Supplementary Materials

The following supporting information can be downloaded at: https://www.mdpi.com/article/10.3390/jcm12062354/s1, Table S1: Demographic and clinical data of PDR patients with tractional retinal detachment or vitreous hemorrhage. Table S2: Vitreous fluid levels of cytokines in PDR patients with tractional retinal detachment or vitreous hemorrhage. Table S3: Correlation matrices with Spearman’s rank correlation coefficients for systemic factors and vitreous fluid cytokines in PDR patients and controls. Figure S1: Disposition of PDR patients.
