# Supplementary material for: Association between Systemic Factors and Vitreous Fluid Cytokines in Proliferative Diabetic Retinopathy
Source: J Clin Med. 2023 Mar 17;12(6):2354. doi: 10.3390/jcm12062354 (PMC10059790; doi:10.3390/jcm12062354)
Supplement: Supplementary file 1 [file jcm-12-02354-s001.zip › jcm-2261403-supplementary.pdf]

Table S1: Demographic and clinical data of PDR patients with tractional retinal detachment or vitreous hemorrhage.

Category	PDR				*p* value	Unit	Reference range
	TRD		VH				
n	3		10				
	Detectable rate (%)	Mean ± SD	Detectable rate (%)	Mean ± SD			
Age	3 (100)	46.3 ± 6.51	10 (100)	59.4 ± 12.6	0.159	year	
Gender (M/F)	3 (100)/0		7 (70.0)/ 3		0.528		
Laterality (R/L)	1/2		5/5		1.00		
LogMAR VA	3 (100)	0.93 ± 0.68	10 (100)	1.71 ± 0.70	0.159		
IOP	3 (100)	13.2 ± 2.57	10 (100)	14.1 ± 3.96	0.555	mmHg	10 to 21
CRT	1 (33.3)	319.0	1 (10)	304.0	0.425	µm	
Subgroup							
PRP（+/－）	2 (66.7)/1		5 (50.0)/5				
Focal PC （+/－）	0 (0)/3		0 (0)/10				
DME（+/－）	2 (66.7)/1		8 (80.0)/2				
TRD（+/－）	3 (100)/0		0 (0)/10				
VH（+/－）	0 (0)/3		10 (100)/0				
BMI	3 (100)	25.1 ± 3.29	10 (100)	23.6 ± 4.58	0.372		18.5 to 25.0
SBP	3 (100)	125.7 ± 4.04	10 (100)	127.9 ± 18.6	0.529	mmHg	140 >
DBP	3 (100)	77.3 ± 3.79	10 (100)	76.6 ± 13.2	0.529	mmHg	90 >
PPD	3 (100)	48.3 ± 0.58	10 (100)	51.3 ± 13.5	0.581	mmHg	
HR	3 (100)	78.0 ± 7.21	10 (100)	81.4 ± 9.62	0.555	beats/min	45 to 85
PT-INR	3 (100)	0.93 ± 0.03	10 (100)	0.98 ± 0.05	0.293		0.85 to 1.15

APTT	3 (100)	25.9 ± 1.48	10 (100)	28.2 ± 2.41	0.159	sec	24 to 34
RBG	3 (100)	165.3 ± 23.4	10 (100)	159.7 ± 75.0	0.529	mg/dL	70 to 109
HbA1c	3 (100)	7.27 ± 0.71	10 (100)	7.85 ± 2.00	0.568	%	4.6 to 6.2
BUN	3 (100)	30.3 ± 25.7	10 (100)	21.8 ± 9.03	0.581	mg/dL	8 to 20
Cre	3 (100)	1.79 ± 1.77	10 (100)	1.25 ± 0.54	0.607	mg/dL	0.65 to 1.07
eGFR	3 (100)	69.7 ± 57.7	10 (100)	51.7 ± 21.0	0.581	mL/min/1.73m^2	≥ 60
CRP	3 (100)	0	10 (100)	0.10 ± 0.32	0.568	mg/dL	0.3 >
U-glucose	3 (100)	1.33 ± 1.53	10 (100)	2.15 ± 1.86	0.503	mg/dL	2 to 20
U-protein	3 (100)	2.17 ± 1.44	10 (100)	0.95 ± 1.04	0.179	mg/dL	0 to 10

PDR subgroup with TRD but no VH (*n* = 3) and PDR subgroup with VH but no TRD (*n* = 10) were extracted, and PDR patients with TRD and VH (*n* = 13) were excluded. Comparisons between the two groups were performed. APTT; activated partial thromboplastin time, BMI; body mass index, BUN; blood urea nitrogen, Cre; creatinine, CRP; C-reactive protein, CRT; central retinal thickness, DBP; diastolic blood pressure, DME; diabetic macular edema, eGFR; estimated glomerular filtration rate, F; female, HbA1c; glycated hemoglobin A1c, HR; heart rate, IOP; intraocular pressure, logMAR; logarithm of the minimum angle of resolution, L; left, M; male, *n*; number, PC; photocoagulation, PDR; proliferative diabetic retinopathy, PPD; pulse pressure difference between SBP and DBP, PRP; panretinal photocoagulation, PT-INR; international normalized ratio of prothrombin time, R; right, RBG; random blood glucose, SBP; systolic blood pressure, SD; standard deviation, sec; second, TRD; tractional retinal detachment, U-glucose; urine glucose, U-protein; urine protein, VA; visual acuity, VH; vitreous hemorrhage.

Table S2: Vitreous fluid levels of cytokines in PDR patients with tractional retinal detachment or vitreous hemorrhage.

Category	PDR				*p* value	Detection range
	TRD		VH			
n	3		10			
	Detectable rate (%)	Mean ± SD	Detectable rate (%)	Mean ± SD		
PDGF-BB	0 (0)	0	2 (20.0)	5.21 ± 15.4	0.529	1.00 to 52401
IL-1β	0 (0)	0	2 (20.0)	0.07 ± 0.15	0.529	0.06 to 4598
IL-1ra	2 (66.7)	7.01 ± 7.79	7 (70.0)	22.2 ± 18.0	0.293	2.52 to 247147
IL-2	0 (0)	0	0 (0)	0	0.607	0.54 to 26397
IL-4	2 (66.7)	0.17 ± 0.18	4 (40.0)	0.19 ± 0.33	0.555	0.08 to 4035
IL-5	0 (0)	0	0 (0)	0	0.607	3.69 to 81206
IL-6	3 (100)	58.6 ± 90.6	8 (80.0)	106.7 ± 133.0	0.607	0.37 to 21699
IL-7	3 (100)	19.0 ± 6.75	10 (100)	22.7 ± 10.5	0.529	0.49 to 41077
IL-8	3 (100)	31.2 ± 27.5	10 (100)	99.8 ± 121.7	0.503	0.75 to 27477
IL-9	0 (0)	0	2 (20.0)	0.82 ± 1.94	0.529	0.92 to 45633
IL-10	0 (0)	0	1 (10.0)	0.13 ± 0.41	0.568	0.74 to 23402
IL-12	0 (0)	0	1 (10.0)	2.82 ± 8.92	0.568	1.23 to 21022
IL-13	0 (0)	0	4 (40.0)	3.37 ± 6.75	0.331	0.32 to 9203
IL-15	0 (0)	0	1 (10.0)	0.69 ± 2.17	0.568	1.62 to 251038
IL-17A	0 (0)	0	1 (10.0)	1.10 ± 3.47	0.568	1.71 to 41194
Eotaxin	3 (100)	23.7 ± 14.3	9 (90.0)	11.9 ± 7.71	0.200	0.07 to 7488
bFGF	0 (0)	0	1 (10.0)	3.33 ± 10.5	0.568	3.02 to 3939
G-CSF	1 (33.3)	5.89 ± 10.2	4 (40.0)	10.8 ± 17.4	0.555	1.67 to 138193
GM-CSF	0 (0)	0	0 (0)	0	0.607	0.38 to 10559
IFN-γ	3 (100)	19.2 ± 9.25	9 (90.0)	31.9 ± 27.6	0.414	0.74 to 20941
IP-10	3 (100)	2948.5 ± 3107.4	10 (100)	4586.2 ± 6595.2	0.555	2.75 to 48834
MCP-1	3 (100)	336.2 ± 93.8	10 (100)	664.1 ± 503.7	0.200	0.44 to 11213
MIP-1α	3 (100)	0.84 ± 0.61	10 (100)	1.73 ± 1.68	0.462	0.05 to 1045

MIP-1β	3 (100)	3.57 ± 1.63	10 (100)	11.2 ± 10.1	0.159	0.29	to	6180
RANTES	0 (0)	0	4 (40.0)	5.74 ± 9.48	0.331	1.41	to	7569
TNFα	3 (100)	11.4 ± 11.3	7 (70.0)	13.9 ± 19.2	0.594	2.73	to	63996
VEGF-A	2 (66.7)	122.8 ± 188.9	7 (70.0)	220.7 ± 225.3	0.462	2.42	to	178228

bFGF; basic fibroblast growth factor, G-CSF; granulocyte colony-stimulating factor, GM-CSF; granulocyte macrophage colony-stimulating factor, IFN; interferon, IP-10; interferon gamma-induced protein 10, IL; interleukin, MIP; macrophage inflammatory protein, MCP; monocyte chemotactic protein, PDGF; platelet derived growth factor, ra; receptor antagonist, RANTES; regulated on activation, normal T-cell expressed and secreted, TNF; tumor necrosis factor, VEGF; vascular endothelial growth factor.

Table S3: Correlation matrices with Spearman's rank correlation coefficients for systemic factors and vitreous fluid cytokines in PDR patients and controls.

Control group

	Age	BMI	SBP	DBP	PPD	HR	PT-INR	APTT	RBG	HbA1c	eGFR	IL-1ra	IL-6	IL-7	IL-8	IL-13	Eotaxin	IFN-γ	IP-10	MCP-1	MIP-1β	TNFα	VEGF-A
**Age**	–	-0.223	-0.004	-0.102	0.080	-0.126	0.295	0.364	0.252	0.154	0.221	-0.234	0.115	0.065	-0.006	0.311	0.265	0.096	0.347	0.120	-0.014	-0.312	-0.011
**BMI**		–	0.127	0.128	0.124	0.032	-0.131	**-0.372**	0.003	-0.048	-0.324	0.003	0.054	-0.022	0.059	0.236	0.043	-0.095	0.067	-0.186	0.059	0.054	-0.097
**SBP**			–	**0.709**	**0.844**	**0.566**	0.112	0.174	-0.198	-0.312	-0.137	-0.109	-0.187	-0.065	0.088	0.005	0.039	-0.049	-0.152	-0.035	-0.138	-0.032	-0.054
**DBP**			**	–	0.320	**0.613**	-0.025	0.172	**-0.473**	**-0.455**	0.157	-0.162	-0.012	-0.041	0.189	0.053	0.229	-0.030	0.018	-0.017	0.016	0.161	-0.204
**PPD**			**		–	0.246	0.038	0.065	-0.014	-0.085	-0.203	-0.013	-0.243	-0.055	0.018	0.086	-0.068	-0.034	-0.199	-0.073	-0.181	-0.204	0.064
**HR**			**	**		–	0.205	0.230	-0.243	-0.326	-0.103	-0.298	0.042	0.025	0.082	-0.226	0.029	0.013	0.002	0.065	-0.004	0.140	-0.183
**PT-INR**							–	**0.488**	0.167	0.045	-0.119	-0.257	-0.189	-0.153	-0.309	0.175	0.000	-0.047	0.065	0.010	0.093	-0.205	-0.205
**APTT**		*					**	–	-0.049	-0.359	-0.028	-0.225	-0.278	0.123	0.034	0.072	0.161	0.183	0.193	0.172	0.128	-0.023	-0.181
**RBG**				**					–	0.328	-0.230	-0.058	-0.125	0.035	-0.071	-0.027	-0.024	0.285	0.170	0.194	0.020	-0.269	-0.183
**HbA1c**				*						–	0.026	-0.023	0.075	-0.141	0.166	-0.062	0.087	0.115	0.210	0.154	-0.352	-0.284	N/A
**eGFR**											–	-0.221	0.037	0.011	-0.151	0.063	0.101	-0.198	0.024	-0.210	-0.232	0.011	-0.204
**IL-1ra**												–	0.180	-0.131	0.003	0.001	**-0.538**	0.128	**-0.433**	0.072	-0.127	-0.154	0.273
**IL-6**													–	0.255	0.331	-0.151	0.265	**0.465**	0.350	**0.537**	**0.397**	-0.118	0.311
**IL-7**														–	0.264	-0.314	**0.487**	**0.459**	**0.593**	**0.362**	0.217	0.097	-0.194
**IL-8**															–	0.013	0.240	**0.388**	0.226	0.344	0.095	0.087	0.271
**IL-13**																–	-0.047	-0.028	-0.211	-0.240	0.089	-0.062	-0.062
**Eotaxin**												**		**			–	0.280	**0.767**	0.274	**0.387**	0.139	-0.290
**IFN-γ**													**	*	*			–	**0.378**	**0.895**	**0.432**	-0.118	0.097

**IP-10**												*		**			**	*	–	**0.423**	**0.390**	-0.097	-0.290
**MCP-1**													**	*				**	*	–	**0.510**	-0.139	0.247
**MIP-1β**													*				*	*	*	**	–	0.107	0.032
**TNFα**																						–	-0.034
**VEGF-A**																							–

PDR group

	Age	BMI	SBP	DBP	PPD	HR	PT-INR	APTT	RBG	HbA1c	eGFR	IL-1ra	IL-6	IL-7	IL-8	IL-13	Eotaxin	IFN-γ	IP-10	MCP-1	MIP-1β	TNFα	VEGF-A
**Age**	–	-0.374	-0.065	**-0.611**	**0.445**	-0.117	0.128	0.162	0.161	-0.264	-0.036	-0.006	-0.229	-0.062	0.379	0.151	-0.381	-0.255	-0.213	-0.114	0.158	-0.243	-0.050
**BMI**		–	**0.438**	0.249	0.234	0.124	0.170	0.062	0.059	0.312	0.059	**-0.392**	**0.400**	0.362	0.020	0.160	0.162	-0.070	**0.439**	-0.049	-0.076	-0.045	-0.199
**SBP**		*	–	**0.540**	**0.604**	0.304	-0.109	-0.179	**0.445**	0.125	-0.117	0.011	**0.467**	**0.465**	0.242	**0.440**	**0.424**	0.290	**0.589**	0.279	0.277	0.244	0.264
**DBP**	**		**	–	-0.260	0.366	-0.224	-0.372	0.178	0.160	-0.208	0.022	**0.459**	0.395	-0.123	0.222	**0.510**	0.319	**0.433**	0.295	0.166	0.287	0.187
**PPD**	*		**		–	0.009	0.030	0.204	0.241	-0.170	0.000	0.048	0.116	0.195	0.283	0.295	0.009	0.035	0.269	0.068	0.131	0.025	0.117
**HR**						–	0.072	-0.058	0.019	0.296	0.044	-0.034	0.375	0.101	0.007	0.049	0.399	0.090	0.390	0.032	0.042	0.278	0.233
**PT-INR**							–	**0.545**	**-0.459**	-0.149	0.333	-0.287	0.116	0.111	-0.086	0.297	0.030	-0.176	0.081	-0.195	-0.174	-0.174	0.080
**APTT**							**	–	-0.243	-0.304	0.309	0.075	0.000	-0.175	0.069	-0.056	-0.146	0.093	0.185	0.114	-0.136	0.169	-0.145
**RBG**			*				*		–	0.299	-0.067	0.132	-0.039	0.039	0.130	-0.147	-0.082	0.082	0.091	0.173	0.131	0.018	-0.147
**HbA1c**										–	0.128	-0.186	0.097	-0.080	-0.184	-0.215	-0.104	0.006	0.081	-0.025	-0.003	-0.130	-0.285
**eGFR**											–	-0.211	-0.088	0.094	0.187	0.251	-0.102	-0.104	0.063	-0.009	0.093	-0.026	0.098
**IL-1ra**		*										–	-0.347	**-0.424**	0.301	-0.260	-0.109	**0.677**	-0.129	**0.612**	0.081	**0.618**	0.195
**IL-6**		*	*	*									–	**0.701**	-0.120	0.355	**0.459**	-0.028	**0.778**	0.025	0.308	-0.087	0.286
**IL-7**			*									*	**	–	0.133	**0.753**	0.228	-0.143	**0.475**	-0.015	**0.394**	-0.183	**0.443**
**IL-8**															–	0.323	-0.119	**0.446**	0.257	**0.584**	**0.592**	**0.406**	0.206
**IL-13**			*											**		–	0.227	-0.020	0.285	0.115	0.350	0.051	**0.574**

**Eotaxin**		*	*									*				–	0.246	**0.524**	0.040	0.014	**0.397**	**0.578**
**IFN-γ**											**			*			–	**0.407**	**0.874**	0.309	**0.823**	0.225
**IP-10**	*	**	*									**	*			**	*	–	**0.449**	**0.465**	0.284	0.236
**MCP-1**											**			**			**	*	–	**0.566**	**0.697**	0.194
**MIP-1β**													*	**				*	**	–	0.079	0.304
**TNFα**											**			*		*	**		**		–	**0.389**
**VEGF-A**													*		**	**					*	–

Numerical data are Spearman's rank correlation coefficients. Asterisks denote significant differences. Blue boxes indicate positive correlation, and red boxes denote negative correlation. N/A; not applicable because correlation coefficient is less than 1.0×10^{-10}, *$p < 0.05$, **$p < 0.01$.

Figure S1: Disposition of PDR patients.

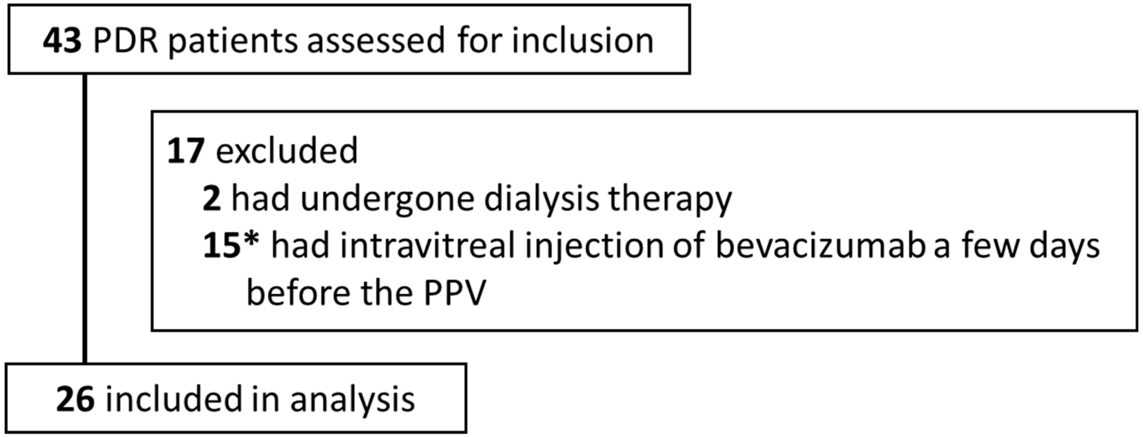

*; Patients received intravitreal injection of bevacizumab prior to undergoing PPV had high disease activity of retinopathy, and were at high risk of developing intraoperative complications such as vitreous hemorrhage. PDR; proliferative diabetic retinopathy, PPV; pars plana vitrectomy.
